# The impact of social anxiety on feedback-based go and nogo learning

**DOI:** 10.1007/s00426-021-01479-5

**Published:** 2021-02-01

**Authors:** Jutta Peterburs, Christine Albrecht, Christian Bellebaum

**Affiliations:** 1 Department of Biological Psychology, Institute of Experimental Psychology, Heinrich-Heine-University Düsseldorf, Universitätsstraße 1, 40225 Düsseldorf, Germany; 2 Department of Medicine, Medical Psychology, MSH Medical School Hamburg, Am Kaiserkai 1, 20457 Hamburg, Germany

## Abstract

The term “Pavlovian” bias describes the phenomenon that learning to execute a response to obtain a reward or to inhibit a response to avoid punishment is much easier than learning the reverse. The present study investigated the interplay between this learning bias and individual levels of social anxiety. Since avoidance behavior is a hallmark feature of social anxiety and high levels of social anxiety have been associated with better learning from negative feedback, it is conceivable that the Pavlovian bias is altered in individuals with high social anxiety, with a strong tendency to avoid negative feedback, especially (but not only) in a nogo context. In addition, learning may be modulated by the individual propensity to learn from positive or negative feedback, which can be assessed as a trait-like feature. A sample of 84 healthy university students completed an orthogonalized go/nogo task that decoupled action type (go/nogo) and outcome valence (win/avoid) and a probabilistic selection task based upon which the individual propensity to learn from positive and negative feedback was determined. Self-reported social anxiety and learning propensity were used as predictors in linear mixed-effect model analysis of performance accuracy in the go/nogo task. Results revealed that high socially anxious subjects with a propensity to learn better from negative feedback showed particularly pronounced learning for nogo to avoid while lacking significant learning for nogo to win as well as go to avoid. This result pattern suggests that high levels of social anxiety in concert with negative learning propensity hamper the overcoming of Pavlovian bias in a win context while facilitating response inhibition in an avoidance context. The present data confirm the robust Pavlovian bias in feedback-based learning and add to a growing body of evidence for modulation of feedback learning by individual factors, such as personality traits. Specifically, results show that social anxiety is associated with altered Pavlovian bias, and might suggest that this effect could be driven by altered basal ganglia function primarily affecting the nogo pathway.

## Introduction

Adaptive behavior, i.e., the optimization of response strategies based on performance-related feedback, is the key to successful survival in dynamic environments. In general, individuals strive to maximize desirable and minimize unfavorable action consequences, as formalized in the Law of Effect by Edward Thorndike (1927). However, not all contingencies between actions and consequences are learned equally well. A growing body of evidence points to the existence of a specific learning bias due to which reward seeking is particularly coupled with action invigoration, while punishment avoidance is particularly coupled with action inhibition (Gray & MacNaughton, 2003). In other words, learning to execute a response to obtain a reward is easier than learning to inhibit a response to obtain a reward, and learning to inhibit a response to avoid punishment is easier than learning to execute a response to avoid punishment (e.g., Guitart-Masip et al., 2012a, 2012b). This bias has been referred to as “Pavlovian bias” and is thought to originate from a conflict between Pavlovian control of behavior, which favors approach in the prospect of reward and avoidance in the prospect of punishment (Gray & MacNaughton, 2003), and instrumental control of behavior, in which the behavioral output depends entirely on outcome valence (Guitart-Masip, Duzel, Dolan, & Dayan, 2014a, Guitart-Masip et al., 2014b). It has been proposed that prefrontal executive control mechanisms are needed to overcome these biases in the learning process (Cavanagh, Eisenberg, Guitart-Masip, Huys, & Frank, 2013). In addition, the hippocampus has also been implicated in processing of approach-avoidance conflicts (for a review, see Ito & Lee, 2016).

Interestingly, the Pavlovian bias is quite robust and evident not only when performing oneself, but also when learning merely by observing another individual’s actions and their consequences (Peterburs, Frieling, & Bellebaum, 2020). Aside from contextual factors, such as agency, a growing number of studies have investigated modulation of feedback-based learning by inter-individual factors. For instance, depression, and specifically anhedonia, i.e., a lack of pleasure in response to ordinarily rewarding experiences, is associated with reduced reward sensitivity (e.g.; Huys, Pizzagalli, Bogdan, & Dayan, 2013; see Must, Horvath, Nemeth, & Janka, 2013 for a mini review). Nevertheless, the Pavlovian bias is preserved in patients with mild to moderate major depressive disorder, suggesting that the motivational deficits typical for this disorder cannot be solely explained in terms of aberrant reward processing, and that an altered Pavlovian bias is unlikely to impair recovery (Moutoussis et al., 2018). In contrast, a very recent study reported that patients with first-episode psychosis were impaired at overcoming Pavlovian bias (as reflected in lower learning rates and overall performance) and showed blunted sensitivity to both reward and punishment (Montagnese et al., 2020), suggesting that these alterations in outcome processing may play an important role for the psychopathology of this disorder.

Social anxiety disorder (SAD), which is characterized by disproportional fear in and of (social) performance situations and feedback, has also been linked to alterations in reward processing and feedback-based learning. Cognitive models of the disorder (Clark & Wells, 1995; Rapee & Heimberg, 1997) postulate information processing biases towards increased attention to and memory for negative information as well as negative interpretation of ambiguous information. In line with this, empirical studies have found that high socially anxious individuals learned better than non-socially anxious individuals to avoid stimuli that were associated with negative feedback (Abraham & Hermann, 2015; Voegler, Peterburs, Bellebaum, & Straube, 2019) or ambiguous stimuli (Stevens, Peters, Abraham, & Hermann, 2014) in probabilistic learning tasks. These studies only used tasks requiring response invigoration. Social anxiety, however, is characterized particularly by avoidance behavior: high socially anxious individuals often do not act, i.e., they do not go out, do not meet others, they avoid giving speeches, etc., and the persistence of this behavior suggests that it is reinforced because (anticipated) negative consequences of social situations are avoided. Moreover, this avoidance also prevents the opportunity for fear extinction, which also contributes to the persistence of the anxiety. It thus seems conceivable that the Pavlovian learning bias is altered in individuals with high social anxiety, with a strong tendency to avoid negative feedback, especially (but not only) by not acting. The present study aimed to investigate this notion. Importantly, previous investigations tested clinical samples (patients with SAD; e.g., Voegler et al., 2019) or extreme groups (Abraham & Hermann, 2015) to characterize the impact of high levels of social anxiety on feedback-based learning. However, it has been proposed that social anxiety is represented on a continuum ranging from subclinical behaviors (e.g., shyness) to clinical manifestation (SAD) based on common underlying dysfunctional mechanisms (Stein, Torgrud, & Walker, 2000). Of note, applying dimensional rather than categorical approaches when studying psychopathology to better understand the full spectrum of mental health and mental illness is also at the heart of the Research Domaine Criteria Initiative (RDoc) of the National Institute of Mental Health. Along these lines, testing extreme groups or patient samples may not be ideal to map the impact of social anxiety as a continuous variable as it occurs in the healthy population. We therefore recruited a large sample of healthy adults who naturally varied in social anxiety (as determined based on self-report). Of note, this sample also included individuals with particularly low or high social anxiety scores but did not include individuals with a clinical diagnosis of SAD.

Subjects completed a variant of the orthogonalized go/nogo task first described by Guitart-Masip et al. (2011) that decoupled action (response execution or inhibition; go/nogo) and outcome valence (win/avoid), thus allowing for direct assessment of the Pavlovian bias. Our main aim was to examine to what extent performance in the orthogonalized go/nogo task was affected by social anxiety. As mentioned above, high levels of social anxiety have previously been associated with better learning from negative than positive feedback (Abraham & Hermann, 2015; Voegler et al., 2019). We therefore also applied the so-called probabilistic selection task that enabled us to assess if subjects generally had a propensity to learn better from positive (approach learning) or negative feedback (avoidance learning). Previous work (e.g., Frank, Seeberger, & O'reilly, 2004, Frank, Woroch, & Curran, 2005; Stocco et al., 2017) demonstrated individual differences in learning from positive and negative feedback in healthy participants which was also reflected in their electrophysiological responses to correct and erroneous stimulus choices, suggesting that the individual propensity to learn from positive/negative feedback may be a trait-like phenomenon that can be directly linked to midbrain dopaminergic function (Frank et al., 2005). We thus included learning propensity as a potential additional predictor that may modulate the Pavlovian learning bias in feedback-based learning.

We expected that higher levels of social anxiety would be associated with reduced Pavlovian bias in a go context due to better learning of go to avoid compared to subjects with lower levels of social anxiety. In contrast, we hypothesized that the Pavlovian bias in the nogo context would be more pronounced in subjects with higher levels of social anxiety, due to better learning of nogo to avoid (compared to nogo to win) relative to subjects with lower levels of social anxiety. These effects of social anxiety on the Pavlovian Bias were expected to interact with learning propensity. Specifically, we expected the hypothesized social anxiety effects (i.e., reduced Pavlovian bias in the go context, more pronounced Pavlovian bias in the nogo context) to be strongest for socially anxious individuals with a strong bias towards better learning from negative than positive feedback.

## Methods

### Subjects

A total of 90 student volunteers (77 women, 13 men) were recruited by public advertisement at Heinrich-Heine-University Düsseldorf, Germany. We aimed to analyze the data with LME models because this approach allows for continuous as well as categorical variables to be entered as predictors (see also “Data analyses” below), and both fixed and random effects can be modelled. For a power analysis, however, an estimate of the effect size would be required, usually taken from previous, related studies. Since, to our knowledge, LME models have not been used to address the present or related research questions, the required sample size was roughly estimated based on a previous study that also used the propensity to learn from positive/negative feedback as predictor, even though the dependent variable was lexical ambiguity resolution (Ceballos, Stocco, & Prat, 2020). However, in this study, 140 participants were assigned to three groups of 38 to 52 subjects according to individual learning propensities, thereby transforming the continuous predictor variable into a categorical one. Since in the present study we exploited the nature of learning propensity and social anxiety as continuous variables, increasing the power, a sample size of 90 was considered sufficient. All subjects had normal or corrected-to-normal vision and were naïve to the study’s intent. Six individuals disclosed a history of a psychiatric disorder when completing a background questionnaire at the end of the test session. Their data were excluded from all analyses, because exclusion criteria were current or past psychiatric or neurological disorders as well as the intake of medication affecting the central nervous system. The final sample thus consisted of 84 subjects (71 women, 13 men) of whom 74 reported to be right-handed and 10 reported to be left-handed. Mean age was 22.5 years (SD 4.5; range 18–37 years).

Written informed consent was obtained from all participants prior to participation. Subjects received course credit for participation. The study conforms to the Declaration of Helsinki and received ethical clearance by the Ethics Board of the Faculty of Mathematics and Natural Sciences at Heinrich-Heine-University Düsseldorf, Germany.

### Assessment of social anxiety

The self-report version of the Liebowitz Social Anxiety Scale (LSAS; Liebowitz 1987) was used to assess the individual level of social anxiety. The LSAS comprises 24 items describing situations that are typically unpleasant for socially anxious individuals. Subjects rate their subjective levels of anxiety and avoidance for each situation on Likert scales ranging from zero (no anxiety/avoidance) to three (severe anxiety/avoidance). Avoidance and anxiety scores are added for the LSAS total score. Mean LSAS score in the present sample was 29.59 (SD 20.08, range 1–91). Since total scores > 60 are commonly found in clinical samples and associated with pathological (generalized) social anxiety disorder (Mennin et al., 2002), the sample included individuals with mild to moderate as well as subjects with severe social anxiety. However, since none of the subjects had a formal diagnosis of social anxiety disorder (SAD), the sample is considered sub-clinical.

### Experimental tasks

#### Assessment of learning from positive and negative feedback with the probabilistic selection task

To assess an individual’s propensity to learn better from positive or negative feedback, which was used as a potential predictor of performance in the orthogonalized Go/NoGo task (see below), a variant of the “probabilistic selection task” first described by Frank et al. (2004) was applied (see also Weismüller et al., 2018). Stimulus presentation and timing was controlled by Presentation software (version 17.2, Neurobehavioral Systems, Inc., Berkeley, CA, USA).

**Fig. 1:**
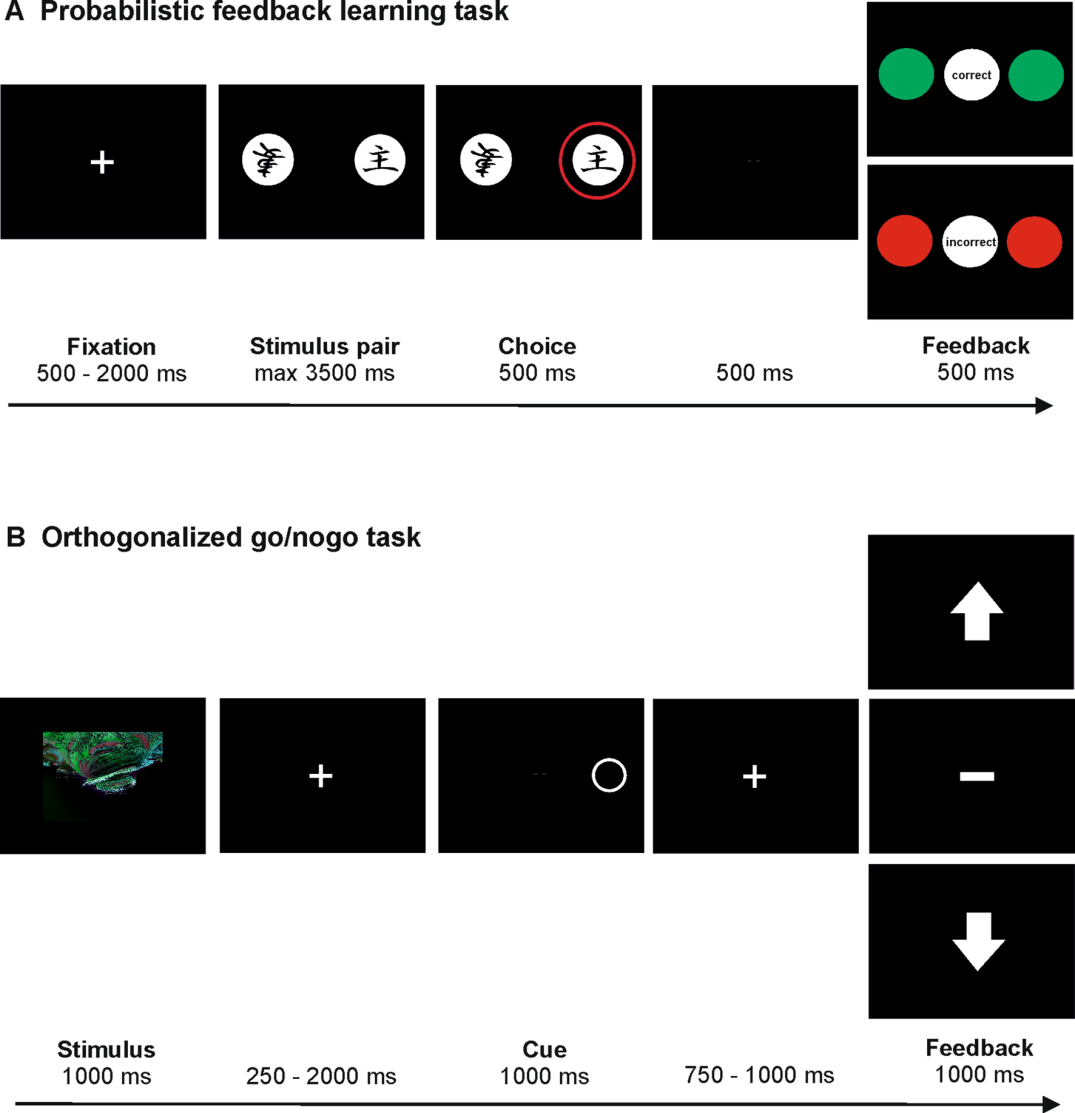
Schematic illustration of the sequence and time course of stimulus presentation in trials in the learning phases of **a** the probabilistic selection task, and **b** the orthogonalized go/nogo task

The task comprised three distinct phases: learning, test, and transfer phase. In the learning phase, in each trial, one of three different pairs of Japanese Hiragana characters was presented (pairs A/B, C/D, and E/F) for up to 3500 ms. Subjects were asked to select one of the characters by pressing the left or right control key on a standard USB keyboard. Upon button press, the selected character was briefly highlighted on the screen for 300 ms before positive or negative feedback was presented for 500 ms, informing the subjects about whether or not their response had been correct. Correct responses were rewarded with + 10 points and incorrect responses were punished with a loss of 10 points towards the total score. Subjects were encouraged to use the feedback to learn which symbols to select to maximize their score. Unbeknown to the subjects, each character was associated with a specific reward probability: for pair A/B, choosing A led to positive feedback in 80% and to negative feedback in 20% of cases. Conversely, choosing B led to positive feedback 20% and to negative feedback 80% of the time. Analogously, the pairs C/D and E/F were associated with reward contingencies of 70/30 and 60/40. If response latency exceeded 3500 ms, the trial was aborted and subjects were reminded to respond faster. Figure 1a illustrates the time course and sequence of stimulus presentation in one trial in the learning phase of the probabilistic learning task. Each pair was presented 20 times in each learning phase, while pair order was randomized, thus amounting to a total of 60 trials per learning phase.

After completion of the learning phase, the test phase started. Here, stimulus sequence and timing were identical to the learning phase except that no feedback was presented. Subjects therefore had to apply the stimulus-outcome associations acquired in the learning phase. To this end, test phases allowed us to determine whether subjects continued to respond according to the knowledge gained during the learning phase in the absence of trial-by-trial feedback (Foerde, Knowlton, & Poldrack, 2006; Foerde & Shohamy, 2011; Kobza et al., 2012). Each pair was presented ten times in each test phase in randomized order, yielding a total of 30 trials per test phase.

In accordance with our previous study (Weismüller et al., 2018), the transfer phase was only started when subjects had reached a fixed learning criterion in the test phase, i.e., once they had chosen correctly in 80% of the trials featuring pair A/B and 70% of the trials featuring pair C/D. If subjects failed to reach this criterion, learning and test phase were repeated. After a maximum of four repetitions (i.e., a total of five learning and five test phases), the transfer phase was initiated regardless of choice accuracy. In the transfer phase, the original pairs were dissolved and the character most likely to result in positive feedback (“A”) was paired with each of the other characters it had not been paired before, yielding the new combinations A/C, A/D, A/E, and A/F. Similarly, the character most likely to result in negative feedback (“B”) was re-paired to yield the new combinations B/C, B/D, B/E, and B/F. This procedure allowed differential assessment of positive and negative learning, i.e., to what extent subjects had learned from positive feedback and thus chosen the stimulus most likely to result in positive feedback (“A”) over all others, and from negative feedback, i.e., to what extent subjects had avoided the stimulus most likely to result in negative feedback (“B”) (e.g., Frank et al., 2004; Kobza et al., 2012; Weismüller et al., 2018). Specifically, the individual propensity to learn from positive/negative feedback was determined as the difference between the percentage of transfer trials in which stimulus A had been chosen and the percentage of transfer trials in which stimulus B had been avoided (e.g., Ceballos et al., 2020). This value could range from − 100 to 100, with negative values indicating better learning from negative and positive values indicating better learning from positive feedback.

The transfer phase consisted of a total of 40 trials, with the stimuli A and B appearing in 20 trials each. No feedback was provided, and stimulus sequence and timing were identical to the test phase.

After each test phase, subjects could take short breaks in which they were informed about their current score. Task completion took between 15 and 40 min, depending on when/if the learning criterion was reached.

#### Assessment of go and nogo learning from positive and negative feedback with the orthogonalized go/nogo task

The second experimental task (again controlled by Presentation software; version 17.2, Neurobehavioral Systems, Inc., Berkeley, CA, USA) was a variant of the “orthogonalized go/nogo task” which decouples outcome valence and action (Guitart-Masip et al., 2011) and is capable of revealing Pavlovian learning biases, which we compared between active and observational feedback learning in a recent study (Peterburs et al., 2020). In the present study, this task assessed the behavior of interest which we hypothesized to be affected by social anxiety, assessed by the LSAS (see “Assessment of social anxiety”), and possibly the propensity to learn from positive or negative feedback, assessed by the probabilistic selection task (see previous section).

In each trial of the task, participants chose between two behavioral options (to execute or inhibit a response, i.e., go or nogo) to receive or avoid losing points. There were four combinations of action and outcome valence (go to win, go to avoid losing, nogo to win, and nogo to avoid losing) and these four options were balanced throughout the task. Four abstract fractal images (Mathôt, Siebold, Donk, & Vitu, 2015; obtained from https://github.com/smathot/materials_for_P0010.5) were used as imperative stimuli and randomly assigned to the four combinations for each subject.

The task consisted of four learning and four test phases which alternated, beginning with a learning block. Learning performance was assessed based on test block performance (see below). Figure 1b illustrates the time course and sequence of stimulus presentation in one trial in the learning phase of the go/nogo task. Trials started with presentation of a fractal image for 1000 ms, followed by a fixation cross for 250–2000 ms. Subsequently, an open circle was presented on the left or right for 1500 ms. Task instructions emphasized that subjects were required to decide between responding and not responding, and in case of responding to press the response button (left or right CTRL key on a standard USB keyboard) corresponding to the side the circle had been presented on (e.g., right CTRL key for circle on right side). Responses had to occur within 1000 ms following stimulus onset. If subjects chose not to respond, they had to let the response period pass. Accidental button presses on the wrong side led to abortion of the trial and subjects being reminded to respond on the side of the circle if they chose to respond. After presentation of the circle, a fixation cross was displayed for 750–1000 ms, followed by symbolic feedback. An upward pointing arrow indicated that ten points had been gained (win), a downward pointing arrow indicated that ten points had been lost (loss), and a horizontal bar indicated that no points had been gained or lost (draw). Based on this feedback, subjects could learn which fractal stimulus was associated with which kind of outcome (win/draw/loss) for which kind of choice (go or nogo). For two fractal images, the favorable outcome was to avoid losing points (draw) and the unfavorable alternative was a loss of points. For two others, a win was the favorable and a draw the unfavorable outcome. For one stimulus per outcome combination, the favorable outcome could be obtained with a go or a nogo choice, respectively. Correct choices led to the favorable outcome in 80% of the trials, while the unfavorable outcome was received in the other 20%.

Similar to the probabilistic selection task, each learning phase was followed by a test phase in which no feedback was provided. Otherwise, test trials were identical to learning trials. Task instructions encouraged subjects to optimize test phase performance based on the feedback provided in the learning phases. Test phases were introduced in the orthogonalized go/nogo task because the assessment of learning from positive/negative feedback in our variant of the probabilistic selection task was also based on trials without feedback (see “Assessment of learning from positive and negative feedback with the probabilistic selection task”). Moreover, in our recent study involving observational learning (Peterburs et al., 2020), we found that the Pavlovian bias is also reflected in participants’ choice behavior in trials without feedback.

In total, the orthogonalized go/nogo task comprised four learning and four test phases with 40 trials each (ten per combination), in randomized order. Subjects could take short breaks between phases in which they were informed about their current score. To keep the subjects motivated and to prevent negative scores especially early on in the task, the starting score was set to 400 points. Completion of this task took approximately 50 min.

### Procedure

All subjects completed the two tasks described above after written informed consent had been obtained and the demographic questionnaire had been filled in. Task order was balanced across the sample to avoid sequence effects.

### Data analyses

Statistical analyses were performed using R (version 3.5.3). As outlined in the Introduction, the focus of the present study was on the impact of social anxiety on the Pavlovian learning bias and on the question if (and how) this bias related to the individual propensity to learn better from positive or negative feedback. In previous studies, relationships between inter-individual factors or personality traits and performance in specific cognitive tasks have been investigated using LME models because both categorical and continuous factors can be included in this type of analysis (e.g., Bellebaum, Ghio, Wollmer, Weismüller, & Thoma, 2020; for a general overview, see Magezi, 2015).

In a first step, we investigated if social anxiety was correlated with the individual propensity to learn from positive/negative feedback. This analysis was done for two reasons: first, previous studies suggested that social anxiety is characterized by a negative learning bias (Abraham & Hermann, 2015; Voegler et al., 2019). However, this result was reported for a clinical sample (Voegler et al., 2019) and for an extreme group of high socially anxious individuals (Abraham & Hermann, 2015), while we aimed to explore if higher levels of social anxiety are associated with a tendency to learn from negative feedback in a non-clinical sample. Second, a potential relationship between social anxiety and the propensity to learn from positive and negative feedback is relevant for the question if both can serve as independent predictors of performance in the orthogonalized go/nogo task.

Next, we set up an analysis on factors influencing the Pavlovian learning bias using the lme4 statistical package (version 1.1-21) in R (R Core Team) largely following the best practice guide for LME model analysis by Meteyard and Davies (2020). Specifically, the dependent variable was response accuracy in the orthogonalized go/nogo task (i.e., the percentage of correct responses on test trials). From within this task, action type (go/nogo) and outcome (win/avoid) were defined as categorical fixed-effect predictors, and block (1–4) was coded as a continuous predictor. In addition, the separately assessed variables social anxiety, (based on self-reports in the LSAS) and propensity to learn from positive/negative feedback (based on performance in the transfer phase of the probabilistic selection task) served as continuous predictors. We aimed to include the latter into the statistical model only if it did not correlate significantly with social anxiety (see above and “Results”) and if it significantly improved the statistical model, as revealed by model comparison (see “Results”). Finally, participants were included as random-effects factor in the analysis. We also included the random slopes of the categorical and continuous predictors by participants. The two action type levels were coded as + 1 for go and − 1 for nogo. Similarly, for outcome, win was coded as + 1 and avoid as − 1. The four levels of the block factor were coded as − 1.5, − 0.5, 0.5, and 1.5. LSAS and learning propensity score measures were mean-centered. As our data set included only one data point for the 16 conditions per participant (i.e., the condition means for two levels of action type, two levels of outcome, and 4 blocks), we decided not to compute a full model that would have contained all subject- and item-level random intercepts, random slopes for all within-subjects effects, and random slopes for interactions completely within-subjects to prevent overfitting. However, to include the largest possible number of random slopes per participant, we included all within-subjects main effects as random effects. In other words, we modeled random intercepts for all of our between- and within-subjects variables, and we also included random slopes for all within-subjects effects. The model was specified as follows:

$$\begin{aligned} {\text{Accuracy}} & \sim {\text{block}} \times {\text{action}}\;{\text{type}} \times {\text{outcome}} \times {\text{LSAS}} \times {\text{learning}}\;{\text{propensity}} \\ & \quad + \left( {1 + {\text{block}} + {\text{action}}\;{\text{type}} + {\text{outcome}}|{\text{subject}}} \right). \\ \end{aligned}$$

The model was estimated using a restricted maximum likelihood approach, as proposed by Luke (2017). The R package lmerTest (version 3.1-0) was applied for evaluating significance in the model using Satterthwaite approximation for the degrees of freedom. Only *p* values below the alpha-level of 0.05 were considered significant. To identify statistical outliers, Cook’s distance was calculated using the R package influence.ME (Nieuwenhuis, Grotenhuis, & Pelzer, 2012), and subjects with a Cook’s distance above the cut-off 4/(*n* − *p* − 1) were excluded.

Significant interactions were resolved with the R package interactions (version 1.1.3; Long 2019), using simple slope analyses and fixed cut-off values for the continuous factors, i.e., LSAS scores and propensity to learn from positive/negative feedback. This procedure enables subdivision into slopes for the mean value of the respective continuous factor plus one standard deviation above and below the mean value. Interactions of more than two factors were resolved in a manual, stepwise manner: we first resolved one factor and checked for significance of the remaining lower-level interactions. If these were found to be significant, the next factor was resolved accordingly.

## Results

### Social anxiety and learning from positive and negative feedback

In one subject, there were technical problems with response recording during the assessment of the propensity to learn from negative and positive feedback, so that data from *n* = 83 were entered into the analysis of a relationship between the learning propensity and social anxiety. On average, subjects completed 2.3 learning and test phases (SD = 1.6; range 1–5) before the transfer phase was initiated. Across the whole sample, the A stimulus was selected on average in 71.75% of the trials of the transfer phase (SD = 20.02; range 15–100%), while the B stimulus was avoided on average in 69.16% of trials (SD = 21.68; range 15–100%), resulting in a mean propensity to learn from positive/negative feedback of 2.59% (SD = 29.05), which did not differ significantly from 0 (*p* = 0.419). Thus, there was no general bias towards learning from positive or negative feedback in the present sample. Importantly, correlation analysis revealed that there was no significant relationship between social anxiety and the propensity to learn from positive or negative feedback (*r* = 0.081, *p* = 0.472).

### Social anxiety, learning from positive and negative feedback and the Pavlovian learning bias in the orthogonalized go/nogo task

Based on Cook’s distance (see above), six further participants were excluded from the analysis of the relationship between social anxiety, the propensity to learn from positive/negative feedback and performance in the orthogonalized go/nogo task, so that this analysis was based on *n* = 77 participants. The lack of a significant relationship between social anxiety and learning from positive/negative feedback as reported in the preceding section ensured that the two parameters can be considered as largely independent and thus both be included in the LME model for predicting performance in the orthogonalized go/nogo task. Furthermore, a model comparison based on a likelihood ratio test using the anova() function in R supported inclusion of learning propensity as a continuous factor, in addition to social anxiety (*χ*^2^_(16)_ = 34.51, *p* = 0.005): the model had a smaller Akaike-Information-Criterion (AIC_(43)_ = 11,600, Log-likelihood = − 5757.20) than the respective model without learning propensity (AIC_(27)_ = 11,603, Log-likelihood = − 5774.50), suggesting that the learning propensity added significantly to explaining the variance of performance in the orthogonalized go/nogo task. Note that for this comparison, the models were recalculated using the maximum likelihood rather than the restricted maximum likelihood approach.

**Fig. 2:**
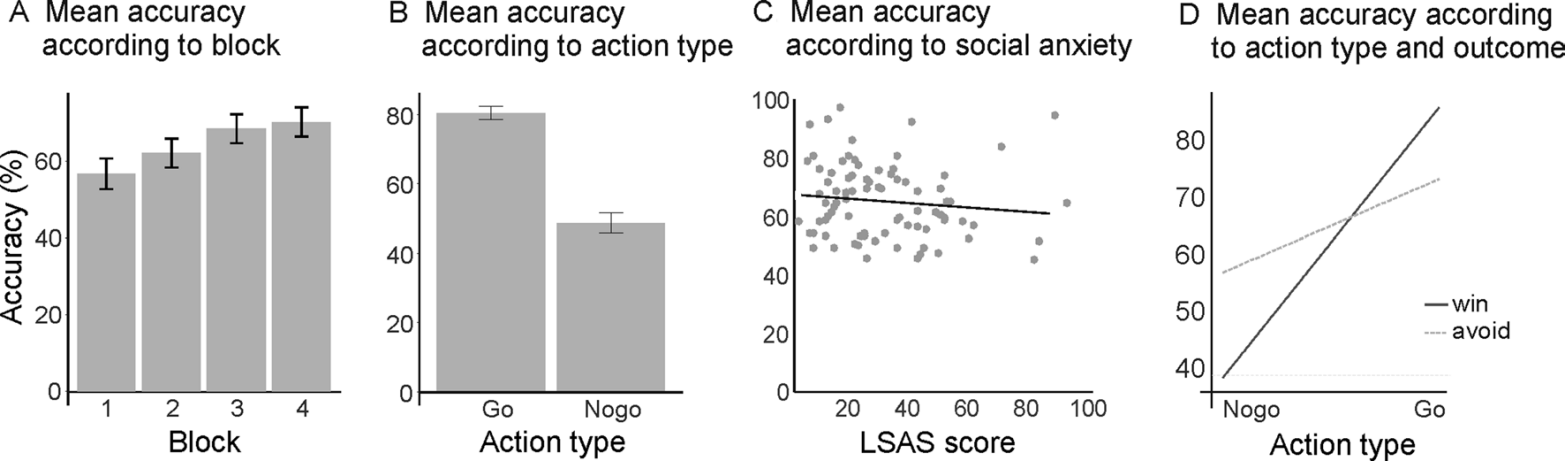
Main effects of block (**a**), action type (**b**), and social anxiety (**c**), and action type × outcome interaction (**d**). Mean accuracy increased across blocks (1–4), was higher for go than nogo, and decreased with increased social anxiety as reflected in LSAS scores. Moreover, accuracy was higher for win than avoid for go trials, while the reverse was true for nogo trials (avoid > win)

Not surprisingly, the LME model revealed significant main effects of block (*F*_[1, 165]_ = 52.67, *p* < 0.001) and action type (*F*_[1, 72]_ = 109.75, *p* < 0.001), indicating that performance generally increased throughout the task (*b* = 4.71) and was better for go than nogo trials (*b* = 15.97). Interestingly, a main effect of social anxiety was also found (*F*_[1, 72]_ = 5.63, *p* = 0. 020): increased social anxiety was associated with decreased performance accuracy (*b* = − 0.19). These effects are illustrated in Fig. 2a–c.

All fixed- and random-effects parameters are provided in Table 1. Importantly, we could replicate the significant action type × outcome interaction from the literature (*F*_[1, 968]_ = 136.68, *p* < 0.001). Simple slope analysis revealed a significant positive slope for the predictor outcome for go trials (*b* = 6.31, *p* < 0.001), indicating that accuracy was higher for win than for avoid. The reverse was found for nogo trials: a significant negative slope (*b* = − 9.25, *p* < 0.001) indicated that accuracy was higher for avoid than for win; see also Fig. 2d). Moreover, we found a number of significant and marginally significant two-, three-, and four-way interactions. These will not be reported in detail, as there was a significant five-way interaction between all factors (block × action type × outcome × social anxiety × learning propensity, *F*_[1, 968]_ = 4.59, *p* = 0. 032). To resolve this interaction, we first considered the factor social anxiety. The subordinate four-way interaction block × action type × outcome × learning propensity was significant for high socially anxious subjects (*F*_[1, 968]_ = 7.42, *p* = 0. 007), but not for low socially anxious subjects (*p* = 0.887). We next resolved the factor learning propensity in high socially anxious subjects. The three-way interaction block × action type × outcome was significant for negative learners among the high socially anxious subjects (*F*_[1, 968]_ = 8.92, *p* = 0. 003), but not for positive learners in this group of participants (*p* = 0.249). As a next step, we considered the factor outcome in high socially anxious negative learners. The subordinate two-way interaction block × action type was significant for avoid (*F*_[1, 968]_ = 13.58, *p* < 0.001), but not for win (*p* = 0.590). Last, we resolved the factor action type. The main effect block was significant for nogo (*F*_[1, 82]_ = 21.69, *p* < 0.001), but not for go (*p* = 0.664).

**Table 1:** Regression table with all fixed and random effect parameters

	*B*	SE	*F*	*P*
Block	4.71	0.65	52.67	< 0.001***
Action type	15.97	1.52	109.75	< 0.001***
Outcome	− 1.48	1.08	1.88	0.175
Social anxiety	− 0.19	0.08	5.63	0.020*
Learning propensity	− 0.02	0.05	0.10	0.748
Block × action type	− 2.38	0.60	15.96	< 0.001***
Block × outcome	− 1.30	0.60	4.78	0.029*
Action type × outcome	7.78	0.67	136.68	< 0.001***
Block × social anxiety	− 0.07	0.04	2.89	0.091
Action type × social anxiety	0.09	0.09	0.93	0.339
Outcome × social anxiety	− 0.13	0.07	4.14	0.045*
Block × learning propensity	0.00	0.02	0.02	0.882
Action type × learning propensity	0.04	0.06	0.60	0.441
Outcome × learning propensity	0.04	0.04	1.35	0.248
Social anxiety × Learning propensity	0.01	0.00	3.10	0.083
Block × action type × outcome	− 0.17	0.60	0.08	0.771
Block × action type × social anxiety	0.00	0.04	0.00	0.998
Block × outcome × social anxiety	− 0.03	0.04	0.54	0.462
Action type × outcome × social anxiety	0.11	0.04	6.94	0.009**
Block × action type × learning propensity	0.00	0.02	0.00	0.948
Block × outcome × learning propensity	0.04	0.02	3.32	0.069
Action type × outcome × learning propensity	− 0.08	0.02	10.57	0.001**
Block × social anxiety × learning propensity	0.00	0.00	1.23	0.269
Action type × social anxiety × learning propensity	0.00	0.00	1.65	0.203
Outcome × social anxiety × learning propensity	0.00	0.00	0.16	0.692
Block × action type × outcome × social anxiety	0.09	0.04	6.44	0.011*
Block × action type × outcome × learning propensity	− 0.05	0.02	4.80	0.029*
Block * action type * social anxiety * learning propensity	0.00	0.00	0.92	0.337
Block * outcome * social anxiety * learning propensity	0.00	0.00	0.38	0.537
Action type * outcome * social anxiety * learning propensity	0.00	0.00	0.08	0.775
Block * action type * outcome *social anxiety * learning propensity	0.00	0.00	4.59	0.032*

**Fig. 3:**
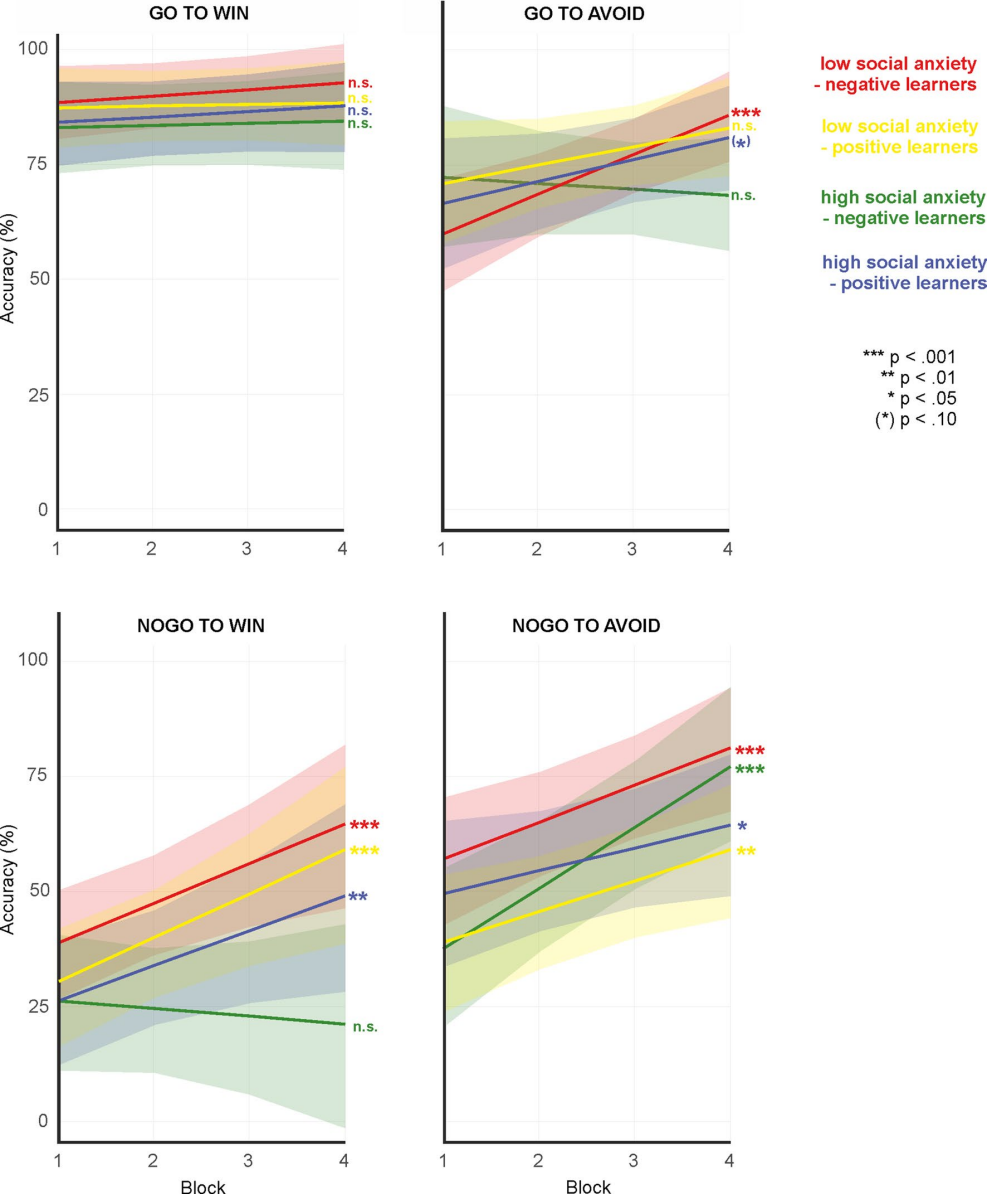
Accuracy as a function of the factors action type (go/nogo), and outcome (win/avoid) according to social anxiety (high/low) and learning propensity (positive/negative learners)

**Fig. 4:**
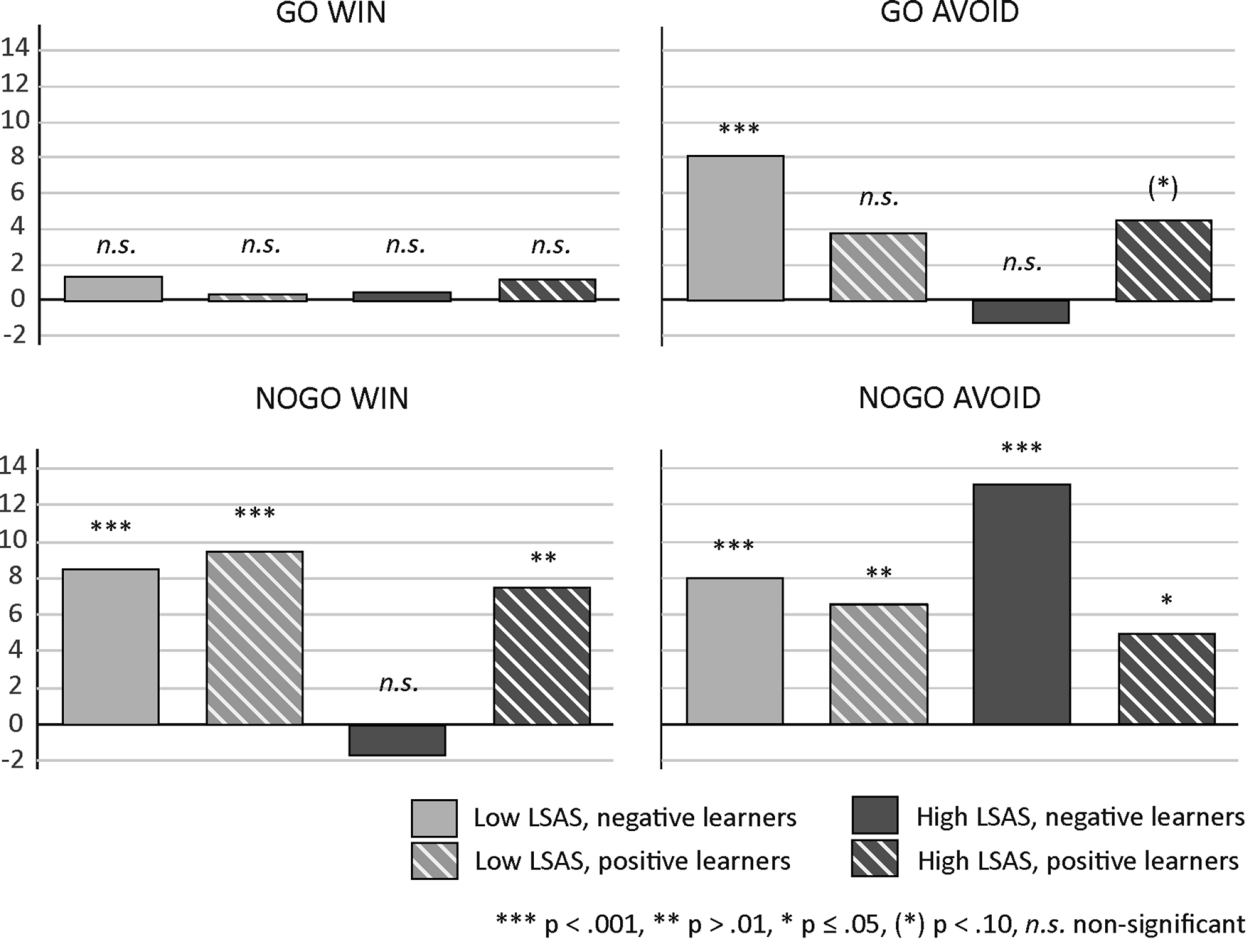
Slope estimates for the factor block as a function of action type (go/nogo) and outcome (win/avoid) according to learning propensity (positive/negative learners) and social anxiety (high/low LSAS score). Note that during the resolution of the 5-way interaction, the effect of the Block factor was calculated separately for each condition. The significance values reported in the Figure refer to these analyses

**Fig. 5:**
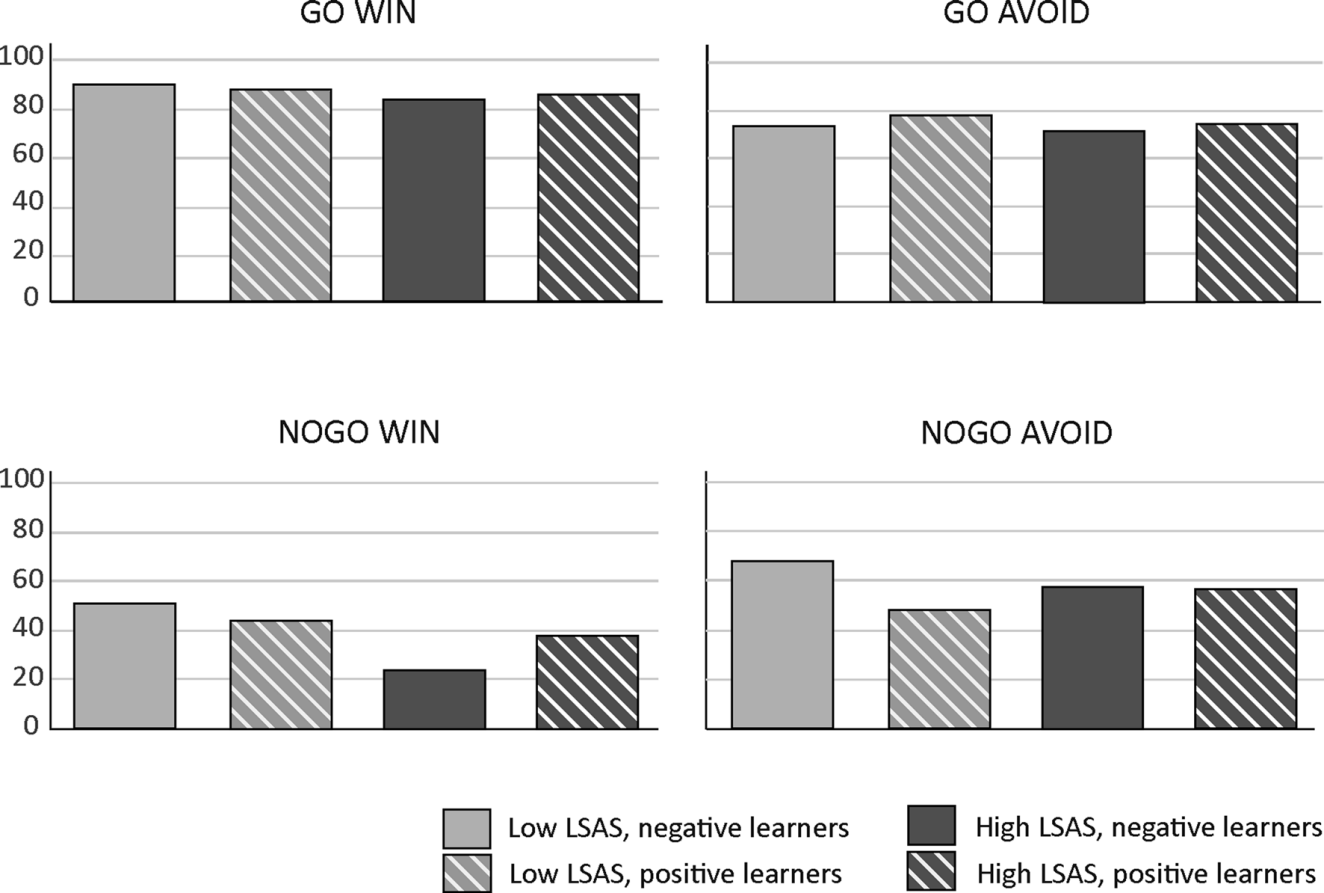
Intercepts for the factor block as a function of action type (go/nogo) and outcome (win/avoid) according to learning propensity (positive/negative learners) and social anxiety (high/low LSAS score)

This result pattern indicates that high socially anxious subjects with a propensity to learn better from negative feedback showed a particularly pronounced increase in accuracy across blocks in the nogo to avoid condition. To fully characterize the result pattern underlying the five-way interaction, we investigated the slopes for the main effect of block also in high socially anxious subjects with a propensity to learn better from positive feedback as well as in low socially anxious positive and negative learners, respectively. Figure 3 provides mean accuracy as a function of block (1–4), action type (go/nogo) and outcome (win/avoid) according to social anxiety (high/low) and learning propensity (positive/negative learners). For illustration purposes, slope estimates and intercepts are provided separately in Figs. 4 and 5. For go to win, no group showed a significant increase in accuracy across blocks, reflecting a ceiling effect. In contrast, nogo to avoid performance was not subject to a ceiling nor a floor effect (see Fig. 2d: average accuracy for nogo to avoid was approx. 40%; see also Fig. 5: intercepts were similar across groups). Here, high socially anxious negative learners showed a particularly high learning rate (i.e., a steeper slope), while lacking significant learning for nogo to win, as well as for the go conditions (see Fig. 4). Importantly, this specific pattern for high socially anxious negative learners in the development of learning across blocks, as reflected in the slope, was not accompanied by a specific pattern in the intercept, which marks the starting point from which performance increase can develop.

## Discussion

The present study investigated the impact of social anxiety on the Pavlovian bias in feedback-based learning, with an additional focus on the individual propensity to learn from positive or negative feedback. To this end, a sample of healthy adults who naturally varied in their levels of social anxiety completed an orthogonalized go/nogo task that decoupled action type (go/nogo) and outcome valence (win/avoid) and a probabilistic selection task based upon which the individual propensity to learn from positive and negative feedback was determined. Self-reported social anxiety and learning propensity were used as predictors in LME model analysis of performance accuracy in the go/nogo task. In line with the expectations, we found an interaction between the Pavlovian bias on the one hand, which is reflected in the interplay of the factors action type (go/nogo) and outcome (win/avoid), the additional factor learning progress across blocks, and the predictor variables social anxiety and learning propensity on the other hand. Specifically, high socially anxious subjects with a propensity to learn better from negative feedback showed particularly pronounced learning for nogo to avoid and lacked significant learning for nogo to win as well as go to avoid. This result pattern indicates that high levels of social anxiety in concert with negative learning propensity hamper the overcoming of Pavlovian bias in a win context while facilitating response inhibition in an avoidance context.

Previous research has confirmed a robust asymmetric interaction of action and outcome type in feedback-based learning: learning to execute a response to obtain a reward or to inhibit a response to avoid punishment is much easier than learning the reverse, a phenomenon referred to as “Pavlovian bias” (Guitart-Masip et al., 2012a, 2012b, 2014a, 2014b; Peterburs et al., 2020). This bias is supported by the present findings. In addition, learning was overall better for go than for nogo, which is also consistent with previous observations (e.g., Guitart-Masip et al., 2014b; Ocklenburg et al., 2017; Peterburs et al., 2020) and could be attributed to higher task difficulty and/or cognitive demand in the context of response inhibition (relative to response execution), or a general propensity to respond in experimental sessions. Also in accordance with previous findings (Peterburs et al., 2020), go to win was an easy condition to learn, as reflected in overall high accuracy rates and a lack of significant learning progress across task blocks. This result pattern strongly suggests a ceiling effect in go to win learning (see also Figs. 3, 4, 5).

Crucially, as expected, we found that both learning propensity and social anxiety together affected performance in the orthogonalized go/nogo task, reflected in an interaction between the five factors block (i.e., learning progress), action type and outcome (reflecting Pavlovian and instrumental conflict), learning propensity (positive/negative learners), and social anxiety. Further analysis revealed that high socially anxious subjects with a propensity to learn better from negative feedback showed a particularly pronounced increase in accuracy across blocks in the nogo to avoid condition, as reflected in a particularly steep slope (see Fig. 3), while at the same time no learning was seen in the other conditions. High socially anxious subjects with a propensity to learn better from negative feedback also eventually achieved a high accuracy level for nogo to avoid, with > 70% in Block 4. This is partly in line with a negative learning bias/better avoidance learning in social anxiety (Abraham & Hermann, 2015; Voegler et al., 2019) and extends these previous findings in showing particularly strong learning of response inhibition to avoid negative feedback. Of note, similar accuracy levels were achieved by low socially anxious subjects with a propensity to learn from negative feedback. Interestingly, high socially anxious subjects with a propensity to learn better from negative feedback also showed a lack of significant learning for nogo to win and also go to avoid. This is somewhat unexpected and somewhat incompatible with the notion of attenuated Pavlovian bias. Rather, the present result pattern appears to suggest that high socially anxious subjects with negative learning bias are impaired in overwriting the Pavlovian bias in a win context. On the other hand, acquisition of response inhibition in context of avoidance is facilitated. In general, these results are consistent with a specific information processing bias in social anxiety that leads to increased attention to and/or memory for negative information (for an overview, see Peschard & Philippot, 2016). Positive feedback, according to SAD psychopathology and cognitive SAD models (Clark & Wells, 1995; Rapee & Heimberg, 1997), is rather unexpected and inconsistent with the self-image of high socially anxious individuals and may thus be processed less efficiently. However, this seems to apply only when a certain degree of task difficulty is reached and (cognitive) resources are taxed, explaining why go to win performance in high socially anxious subjects with negative learning propensity was comparable to that of the other groups in the present study.

It has to be noted that the present sample was subclinical and did not include any individuals with a clinical diagnosis of SAD. Nevertheless, some individuals did report moderate to high social anxiety levels that can typically be observed in clinical samples (LSAS scores > 60; Mennin et al., 2002; Rytwinski et al., 2009). In contrast to previous investigations (e.g., Abraham & Hermann, 2015; Pittig, Pawlikowski, Craske, & Alpers, 2014), the present study was based on a rather large naturalistic sample and did not include a comparison of extreme groups of high and low socially anxious individuals. LME model analysis allowed inclusion of social anxiety as a continuous predictor and revealed that higher levels of social anxiety were associated with generally decreased learning performance. While this is certainly an intriguing result, it must be stressed that the clinical implications remain rather unclear. Future studies should specifically address the Pavlovian learning bias in patients with SAD. The use of disorder-specific stimulus material, such as faces or otherwise social feedback (rather than abstract stimuli), might be particularly informative in this regard and also increase ecological validity. And even outside of clinical populations, the type of feedback may be an interesting factor to manipulate in future experiments. A recent study involving a card gambling task (Case & Olino, 2020) found that monetary and social positive and negative feedback both led to comparable learning (i.e., decreases in plays on disadvantageous decks across the task). Importantly, performance on the task with social feedback was associated with fun-seeking and depressive symptoms, indicating that using different types of feedback may help to better characterize social avoidance learning.

Recent findings have linked both pronounced positive and negative learning biases to better access to subordinate word meanings in a lexical ambiguity priming task (Ceballos et al., 2020). This result suggests that basal ganglia function, which is reflected in these learning biases, directly impacts behavioral flexibility. In the context of the present task, increased behavioral flexibility could be expected to be linked with attenuated Pavlovian bias which would be reflected in more efficient learning of go to avoid as well as nogo to win. However, contrary to this notion, learning propensity only influenced task performance as a function of social anxiety. Of note, there was no population level bias towards positive or negative learning in the present sample and most subjects presented with mild to moderate biases in either direction. This is consistent with previous findings in healthy adults (Frank et al., 2005). There also was no correlation between learning bias and social anxiety, indicating that there is no linear relationship between the severity of social anxiety symptoms and increased learning from negative feedback.

Interestingly, individual differences in learning from positive and negative feedback have been linked to basal ganglia dopaminergic function and underlying genetic differences. Consistent with findings showing that go learning in the context of positive feedback relies particularly on striatal dopamine D1 receptors, a polymorphism in the DARPP-32 gene, which has been associated with D1 receptor effects in synaptic plasticity, was linked to a positive learning bias (Frank, Moustafa, Haughey, Curran, & Hutchison, 2007). Similarly, nogo learning in the context of negative feedback was linked to the C957T polymorphism of the DRD2 gene, a gene associated with striatal D2 receptor function (Frank et al., 2007). Unfortunately, the current study was purely behavioral and thus cannot directly inform about gene or brain level effects. However, based on the present results we could speculate that high levels of social anxiety might be associated with altered basal ganglia function in response to negative feedback primarily affecting the nogo pathway and thus D2 receptor function. More research is needed to directly investigate this notion.

It has to be noted that the present study tested a rather homogenous sample both with regard to age and educational attainment, so findings may not generalize to a (more diverse) community sample. Moreover, subjects were not recruited to balance sex. Sex differences in learning from positive and negative feedback have been reported, with females showing better learning from positive feedback than males (Evans & Hampson, 2015), and may thus present a possible confound in the present study. To our knowledge, potential sex differences with regard to learning to execute or inhibit a response to obtain a reward or avoid punishment have not been investigated yet. A substantial body of literature has explored sex differences in anxiety and mood disorders. With regard to social anxiety, women are more likely than men to have SAD and present with more severe symptoms (e.g., Asher & Aderka, 2018), although findings regarding functional impairment are inconclusive (Asher, Asnaani, & Aderka, 2017). Since our sample included only 17 men, meaningful subgroup analysis elucidating possible sex differences in the impact of social anxiety and learning propensity cannot be performed. However, this may be an interesting avenue for further research, as might be potential effects of acute stress, which has been shown to decrease learning from negative (but not from positive) feedback (Petzold, Plessow, Goschke, & Kirschbaum, 2010).

To conclude, the present study investigated the interplay between individual levels of social anxiety and biases in feedback-based learning. The results confirmed a robust Pavlovian bias: learning to execute a response to obtain positive feedback was easier than learning to inhibit a response to obtain positive feedback, and learning to inhibit a response to avoid negative feedback was easier than learning to execute a response to avoid negative feedback. Importantly, as expected, this asymmetric coupling of action and outcome valence was modulated as a function of social anxiety and individual learning propensity. High socially anxious subjects with a propensity to learn better from negative feedback showed particularly pronounced learning for nogo to avoid and a lack of significant learning for nogo to win as well as for go learning. Thus, high levels of social anxiety in concert with negative learning propensity appear to interfere with the overcoming of Pavlovian bias in a win context while facilitating response inhibition in an avoidance context. In general, these findings add to a growing body of evidence for altered outcome processing and adaptive behavior in social anxiety. It could also be speculated that they might yield interesting clinical implications although the present study did not test a clinical sample. Possibly, determining an individual’s learning propensity and taking into account differences in learning as a function of outcome and action type might help to develop therapeutic interventions targeting SAD.

## Data Availability

The datasets generated during and/or analyzed during the current study are available from the corresponding author upon reasonable request.
